# Non-Transecting Urethroplasty for Bulbar Urethral Strictures—Narrative Review and Treatment Algorithm

**DOI:** 10.3390/jcm11237033

**Published:** 2022-11-28

**Authors:** Nathaniel Coddington, Margaret Higgins, Abrar Mian, Brian Flynn

**Affiliations:** 1 Division of Urology, University of Colorado Anschutz Medical Campus, Denver, CO 80045, USA; 2 College of Osteopathic Medicine, Midwestern University, Downers Grove, IL 60515, USA

**Keywords:** bulbar urethral stricture, non-transecting urethroplasty, sexual dysfunction, algorithm

## Abstract

The bulbar urethra is the most common site of stricture disease for which urethroplasty remains standard of care. A decrease in trauma as an etiology in the developed world and concerns regarding sexual dysfunction related to transection of the corpus spongiosum have placed a renewed emphasis on non-transecting urethroplasty techniques. Here, we present our surgical algorithm with emphasis on non-transecting techniques for bulbar urethral stricture disease and review the current state of literature comparing transecting to non-transecting approaches in order to provide guidance to practitioners on patient selection, counseling, and technique.

## 1. Introduction

The incidence of urethral stricture in the United States is as high as 0.6% with an annual healthcare cost of 191 million, which is expected to increase as the population ages [1]. Urethral stricture refers to the pathological narrowing of the urethral lumen secondary to scar formation in the subepithelial connective tissue and can occur anywhere along the anterior urethra, the portion distal to the genitourinary diaphragm that is enveloped in corpus spongiosum. Within the anterior urethra, the bulbar urethra is the most common site for stricture development, comprising almost 50% of all urethral strictures treated, with the majority in the United States being of either iatrogenic or idiopathic in origin [2,3]. Urethroplasty remains the gold standard in management of this complex disease and has been an area of active innovation since the first urethroplasty in the late 19th century. This review will cover some of the new urethroplasty techniques and how they incorporate into existing treatment pathways. We will focus on non-transecting techniques that preserve blood supply, tissue, and minimize morbidity.

## 2. Diagnosis and Evaluation

Initial evaluation starts with a focused genitourinary history, patient reported outcome index, uroflowmetry, post-void residual, and urinalysis. If the patient has been managed with intermittent self-catheterization or indwelling urethral catheter, a SPC should be placed to allow for urethral rest. Radiologic imaging—retrograde urethrogram (RUG) and/or voiding cystourethrogram (VCUG)—is critical to defining stricture length, location, lumen, as is cystourethroscopy to assessing tissue quality and ruling out malignancy [4].

The surgeon should stage the stricture by length, lumen, and location prior to a surgical intervention. These factors, in addition to patient history, stricture etiology, and patient goals are key in the medical decision making

## 3. Management Options and Treatment Algorithm for Bulbar Urethral Strictures

While initial endoscopic management for appropriately selected patients can achieve short-term success, repetitive treatment is usually unsuccessful [5,6]. Long-term outcomes are poor, with approximately 50% of patients failing at 1 year, and durable cure achieved in less than 30% [7,8]. Current guidelines favor urethroplasty in managing urethral strictures and only indicate dilation or direct vision internal urethrotomy (DVIU) in strictures less than 2 cm in length, or in situations where the increased anesthesia requirement, cost, and higher morbidity of urethroplasty outweigh its higher success rate [9]

**Figure 1:**
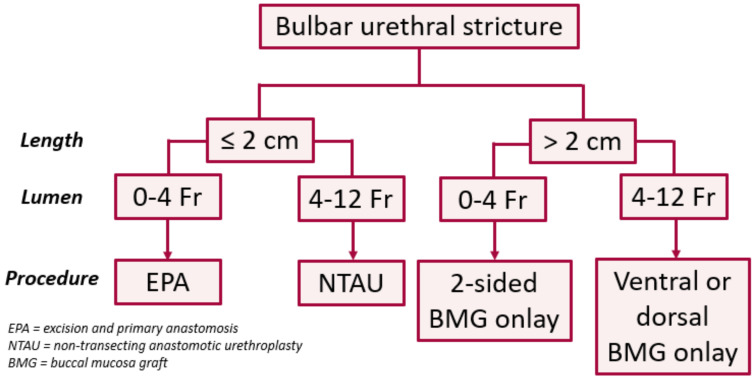
Treatment algorithm for bulbar urethroplasty.

Although a more invasive and technically challenging, urethroplasty has been shown to have superior outcomes when compared to endoscopic procedures, with a long-term success rate of 80–95% [2,10,11]. In the bulbar urethra, the authors use a treatment algorithm based on length and lumen caliber (Figure 1) [12]. In patients with a short (≤2 cm) obliterative (0–4 Fr) strictures, excision and primary anastomosis (EPA) is our treatment of choice, especially for traumatic strictures with severe spongiofibrosis. If the stricture is short but non-obliterative (>4 Fr), we prefer a non-transecting anastomotic urethroplasty (NTAU). In patients with longer (>2 cm) strictures, we prefer non-transecting techniques using buccal mucosa graft (BMG) onlay, ac 1-sided dorsal graft if the stricture lumen is >4 Fr and a two-sided (ventral and dorsal) graft if <4 Fr. While flexible, we prefer the dorsal approach in most circumstances. The 4 Fr cut-off employed here is based on our clinical experience with passage of a standard open-ended catheter into the bladder at the beginning of the case.

## 4. Bulbar Urethroplasty: Transecting vs. Non-Transecting Techniques

For bulbar urethral strictures, an EPA urethroplasty has been the gold standard, with a well-established, durable success rate of 90–95% in properly selected patients [13,14]. Traditional EPAs require full transection of urethra and bulbospongiosus. The scar and surrounding fibrosis are completely excised, and both ends of the urethra are spatulated and reconnected in a tension free manner. The antegrade flow from the bulbourethral arteries is often interrupted. The distal aspect of the urethra then relies partially on retrograde flow from the dorsal penile artery via connections within the glans.

While the stricture patency rates are excellent, concerns have been raised regarding post-operative sexual dysfunction related to both neural and vascular disruptions associated with mobilization and transection of the bulbar urethra [15]. This is not always well captured on existing patient reported outcomes, such as the International Index of Erectile Function (IIEF-5). A recent randomized trial addressed this gap by developing a novel outcome measure specific to urethroplasty with which they were able to detect significant differences in penile complications, including shortening and reduced glans filling, despite similar IIEF-5 scores between transecting and non-transecting groups (Table 1) [16].

Another concern around traditional EPAs relates to the evolving etiology of stricture disease. Iatrogenic strictures related to treatment of prostate hypertrophy and cancer are on the rise [21]. Radiation in particular can be challenging to reconstruction, and roughly 13% of patients will have a stricture recurrence after an EPA in this setting [22]. Limiting dissection in the radiated field is important in order to preserve blood flow for any subsequent procedure for continence, recurrence, or metachronous stricture [23].

In contrast, non-transecting techniques encompass a variety of maneuvers for managing bulbar urethral strictures without full transection of the corpus spongiosum. Grafting techniques, in which the lumen is opened and augmented with buccal mucosa can be thought of as a non-transecting technique, although they were not specifically developed as such [24]. Over the past 15 years, tissue-sparing approaches have been explicitly developed to address the perceived short-comings with EPA. In 2007, Jordan et al., introduced a vessel sparing (VS-EPA) technique designed to preserve proximal blood flow from the bulbourethral arteries in post-prostatectomy patients in order to prevent erosion of a future artificial urinary sphincter or male sling [25]. This was further refined by Mundy in 2012 and has become an invaluable tool in reconstructive armamentarium [23]. An international collaboration demonstrated the long-term patency of this technique; 95% of the 68 patients who underwent a VS-EPA remained patent at 17 month follow-up [26]. It has been shown that patients undergoing VS-EPA had a lesser negative impact on their erectile function compared to those undergoing EPA [18].

## 5. Surgical Technique and Intra-Operative Decision Making

When performing a non-transecting urethroplasty (NTU), surgical dissection is kept to a minimum. Ventrolateral dissection is minimized in order to protect the bulbar arteries. Attempts to preserve the urethral artery within the corpus spongiosum are made by incising the urethra dorsally, though in cases of deep spongiofibrosis, the urethral artery might be compromised. While pre-operative planning with RUG to determine the stricture characteristics guides the surgical technique in most instances, intraoperative findings may alter the surgical needs. For this reason, the authors start with 1-sided dissection to access the dorsal urethra in most cases in efforts to minimize trauma to the local tissue and maintain surgical options.

The patient is placed in a low lithotomy position (no more than 90 degrees of hip flexion). A flexible cystoscope is used to bypass the stricture with a sensor wire, followed by a 5-Fr open ended catheter. A midline perineal incision is made and carried down to the bulbospongiosus muscle. The bulbar urethra is then un-roofed from its surrounding ventral attachments and mobilized on one side from its lateral and dorsal attachment to the tunica albuginea of the corpora cavernosa. Typically, the dissection borders are proximally to the perineal membrane and distally towards the penile suspensory ligament. This is done unilaterally to reduce tissue trauma. A dorsal longitudinal stricturotomy is performed until a 20-French Bougie can be easily passed proximally and distally within healthy appearing urethral mucosa. Stay stitches are used to facilitate visualization and rotate the urethra into the surgical field. At this time, the full length of stricture and degree of spongiofibrosis is determined, which will dictate the surgical technique. Tissue mobility, additional urethral length for spatulation if transection is being considered, as well as the extent of clinical significant fibrosis to be addressed during the procedure are all important to consider.

If the stricture is short (<2 cm), decision making is driven by lumen, degree of spongiofibrosis, and location within the bulbar urethra. Short strictures with minimal underlying fibrosis located within the proximal bulbar urethra can be managed with a stricturoplasty using the Heineke–Mikulicz (HM) principle by closing the longitudinal stricturotomy in a transverse manner [27]. Good urethral mobility and elasticity are critical for the success of this technique. For short strictures with moderate underlying fibrosis, a non-transecting excision of the stricture and mucosal anastomosis, as described by Mundy et al., can be performed [23,28]. This involves excising the abnormal mucosa and underlying spongiofibrosis with preservation of healthy ventral sponge, thus minimizing disruption in longitudinal blood flow. The mucosal ends are then anastomosed together and the dorsal stricturotomy is closed in a transverse manner. If there is an obliterative stricture with significant circumferential spongiofibrosis, as is often the case for traumatic bulbar strictures, excision of the obliterative segment is typically required. If the excised segment is short such that the ends of the urethra approximate without tension, an EPA can be performed in the usual manner. If the excised segment is too long for direct suture closure, an augmented anastomotic urethroplasty (AAU) is performed in which the ventral or dorsal urethra is approximated, and the opposite side is grafted. To perform this in a non-transecting manner (NTAU), a double graft or mucosectomy is required (see below).

**Figure 2:**
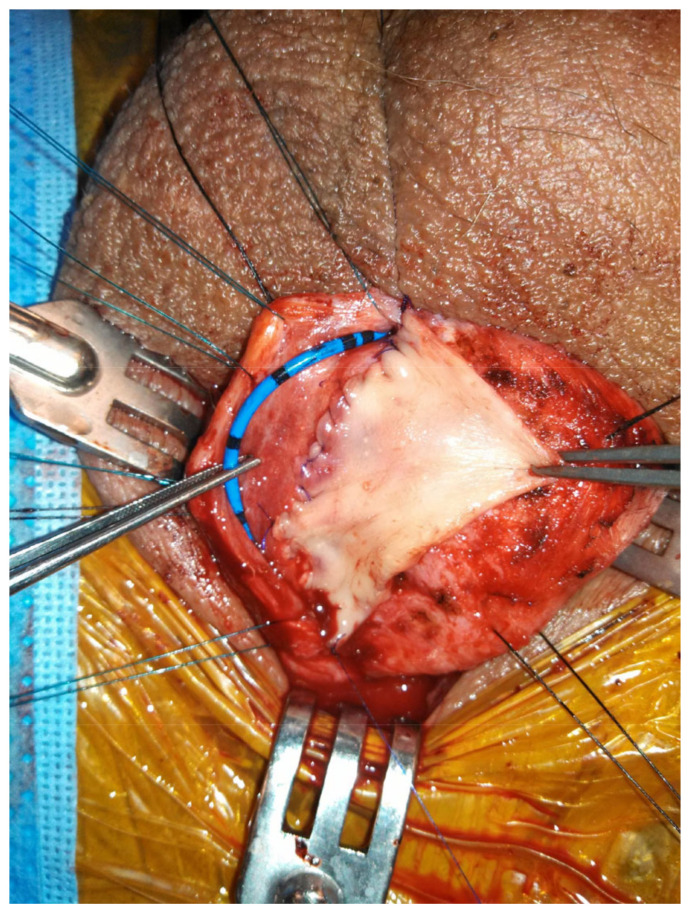
Non-transecting, dorsal onlay buccal mucosal graft urethroplasty.

When the bulbar stricture is long (>2 cm) and non-obliterative, a dorsal onlay urethroplasty can be performed. Initially described by Barbagli in 1996, the technique has been modified over the years and has been shown to have durable success rates [29]. Dorsal graft placement has the benefit of strong corporal fixation, thus reducing risk of sacculation (Figure 2). There is also less operative blood loss due to the thinner bulbospongiosus dorsally. Ventral onlay described by Morey and McAnnich is also an option with durable success [30]. This option is preferred by some when the exposure is challenging due to proximal stricture location or obesity.

**Figure 3:**
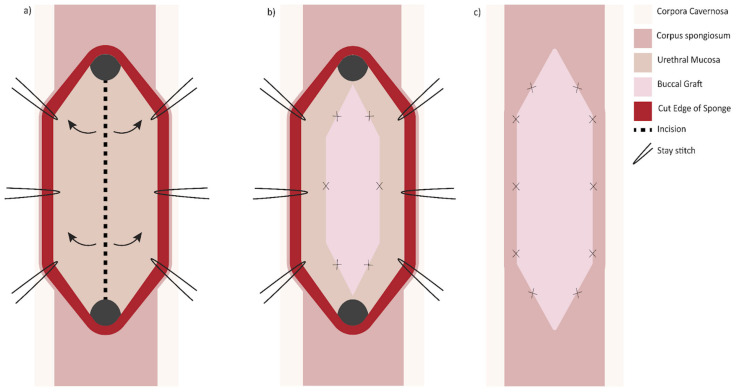
Double graft technique, ventral approach: (**a**) the ventral surface of the urethra is opened and incision made in the dorsal mucosa; (**b**) a buccal graft is sutured in the dorsal defect; (**c**) the ventral urethrotomy is closed with the aid of a second buccal mucosa graft.

**Figure 4:**
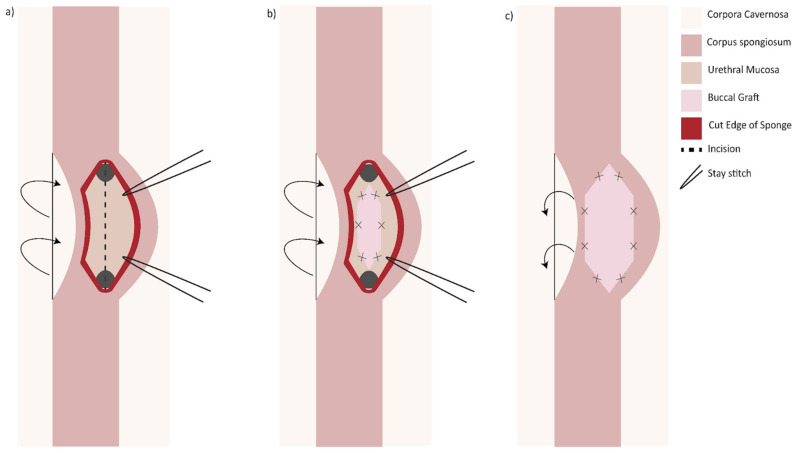
Double graft technique, dorsal approach: (**a**) the urethra is mobilized off the corpora and the dorsal surface is opened. An incision made in the ventral mucosa; (**b**) a buccal graft is sutured in the ventral defect; (**c**) the dorsal urethrotomy is closed with the aid of a second buccal mucosa graft. The urethra is tacked back into position.

Another option when presented with a long (>2 cm) narrow (<4 Fr) stricture is two-sided (dorsal and ventral) grafting technique. The first iteration described by Palminteri in 2008 was performed by making a ventral urethrotomy through which the dorsal urethra is incised to expose tunica albuginea. A dorsal inlay BMG is then placed, followed by a ventral onlay (Figure 3) [31]. Similarly, a dorsal urethrotomy is made through which the ventral urethral mucosa is incised, and a BMG may be inlayed followed by a dorsal onlay (Figure 4). This has the proposed benefit of minimizing trauma to the thicker ventral spongiosum and providing a backing for the ventral graft [32].

## 6. Discussion

Formal comparison of patency rates between urethroplasty techniques is challenging, as most published studies are retrospective cohorts and thus confounded by non-random patient selection for a given approach. The situation is further complicated by the varying definitions of “success” used and length of follow-up, which can affect the true efficacy of urethroplasty and makes comparisons between studies difficult (Table 1) [33]. The available data broadly suggest similar patency rates between transecting and non-transecting techniques in the bulbar urethra [17,18,19]. Barbagli et al., performed a survey of seminal papers on varying bulbar urethroplasty techniques and found that transecting techniques (EPA and AAU) provided success rates ranging from 90 to 98.6% in 404 patients. The non-transecting techniques (NTAU and BMG augmented techniques) provided approximately the same success rate, ranging from 81.8 to 100% in 522 patients [34]. Other groups have also found no significant difference in long-term stricture-free recurrence rates between the transecting and non-transecting bulbar urethroplasty [19]. Important randomized control data awaits publication of the VeSpAR trial, a prospective, interventional, multi-center study with 1:1 randomization of patients to vessel-sparing to transecting anastomotic repair in short bulbar urethral strictures, although this will still leave unanswered questions regarding augmented techniques [35].

Changes in sexual function following urethroplasty has been the subject of intense scrutiny, with mixed results and similar caveats regarding data quality as discussed above. When measured by IIEF-5 or Sexual Health Inventory for Men (SHIM), multiple retrospective studies have shown transient erectile dysfunction (ED) that resolves within 6 months, and little to no difference between transecting and non-transecting groups [15,17,20,36]. Nevertheless, many of these “negative” studies have shown non-significant trends favoring non-transection, and the most recent and largest of these cohorts by Chapman et al., did find significantly more de novo ED in the transecting group at the 6 month mark (14.3% vs. 4.3%, *p* = 0.008) [18]. Further, a 2022 randomized control trial published by Nilsen et al., showed significant increased complaints of penile shortening and decreased glans fullness during erections, albeit with similar IIEF-5 scores between the groups at 3 months and 12 months [16]. This is consistent with prior retrospective data showing increased complaint of penile shortening associated with EPA [17].

Our understanding of bulbar urethral strictures continues to evolve to vessel sparing, non-transecting techniques in efforts to avoid sexual side-effects without compromising surgical outcome. New advancements in surgical technique over the past 15 years have expanded the surgical armamentarium regarding bulbar urethroplasty. The etiology, length, location, and lumen caliber are key criteria to consider when planning surgical repair. Our algorithm provides a logical framework to guide surgical decision making, while still allowing flexibility in procedure selection based on surgical findings. In the setting of a non-traumatic, non-obliterative stricture, the technique should be non-transecting when possible, based on emerging data suggesting improved sexual outcomes with similar patency rates.

## Figures and Tables

**Table 1:** Transecting vs. Non-transecting Bulbar Urethroplasty.

Paper	Year	No. Pts	Study Design	Technique	Primary Outcome	Outcome Measure	Main Result
Nilsen et al. [16]	2022	126	RCT	EPA vs. Graft Onlay	ED Penile complications	IIEF-5 Novel Penile PROM	More penile complication in transecting group, similar rates of ED
Furr et al. [17]	2019	179	Retrospective Cohort	EPA vs. Dorsal Onlay	Recurrence Rate (Early vs. Late) ED LUTS EJD Penile complication	Cystoscopy (Early) and LUTS (Late) IIEF-5 AUA SS MSHQ–EjD Novel PROM	Similar short and long term patency, ED, and EjD. More post-void dribble in onlay and more penile tethering for EPA.
Chapman et al. [18]	2019	352	Retrospective Cohort	EPA vs. NTAU	Recurrence Rate (Early vs. Late) ED Surgical complication	Cystoscopy IIEF-5 Clavien Dindo > 2	Similar patency rates with more de novo ED in EPA group
Anderson et al. [19]	2017	342	Retrospective Cohort	EPA, AAU vs. NTAU, Graft Onlay	Recurrence Rate	Secondary Procedure	Similar stricture free recurrence rates at mean FU of 65 months (83% vs. 82%)
Haines & Rourke [20]	2017	87	Prospective Cohort	EPA vs. NTAU, Graft Onlay	ED	IIEF-5 Adverse Change (>5 pt drop in IIEF-5)	No change in mean IIEF score; non-significant trend favoring non-transection in adverse change (31.8 vs. 16.9%)

## Data Availability

Not applicable.

## Abbreviations

AAU	augmented anastomotic urethroplasty
BMG	buccal mucosa graft
DVIU	direct vision internal urethrotomy
ED	erectile dysfunction
EPA	excision and primary anastomosis
HM	Heineke-Mickulicz
IIEF-5	international index of erectile function
NTAU	non-transecting anastomotic urethroplasty
NTU	non-transecting urethroplasty
RUG	retrograde urethrogram
SPT	suprapubic tube
VCUG	voiding cystourethrogram
VS-EPA	vessel sparing excision and primary anastomosis
